# 432. Intended and Unintended Consequences of a Blood Culture Bottle Shortage: Changes in Antibiotic Prescribing, Contamination Rates, and Sepsis Measures at a Large Academic Institution

**DOI:** 10.1093/ofid/ofaf695.145

**Published:** 2026-01-11

**Authors:** Nicole Kusnik, Juan M Teran Plasencia, Trevor C Van Schooneveld, Jonathan H Ryder

**Affiliations:** University of Nebraska Medical Center, Omaha, NE; University of Nebraska Medical Center/Division of Infectious Diseases, Omaha, NE; University of Nebraska Medical Center, Omaha, NE; University of Nebraska Medical Center, Omaha, NE

## Abstract

**Background:**

We implemented mitigation measures to decrease blood culture (BCx) usage due to a national shortage and sought to explore the association between the blood culture shortage and antibiotic use, contamination rates, and sepsis core measures.

**Image 1:**
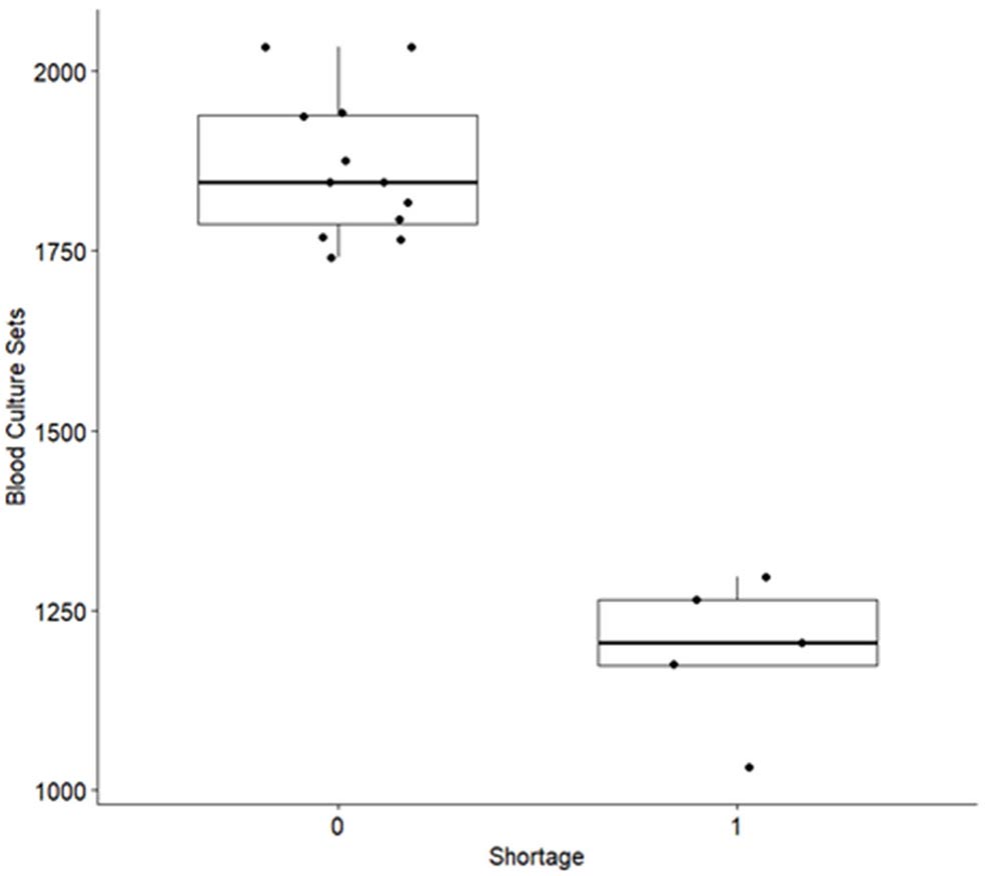
Box plots of total number of blood cultures drawn for the compared time periods

**Image 2:**
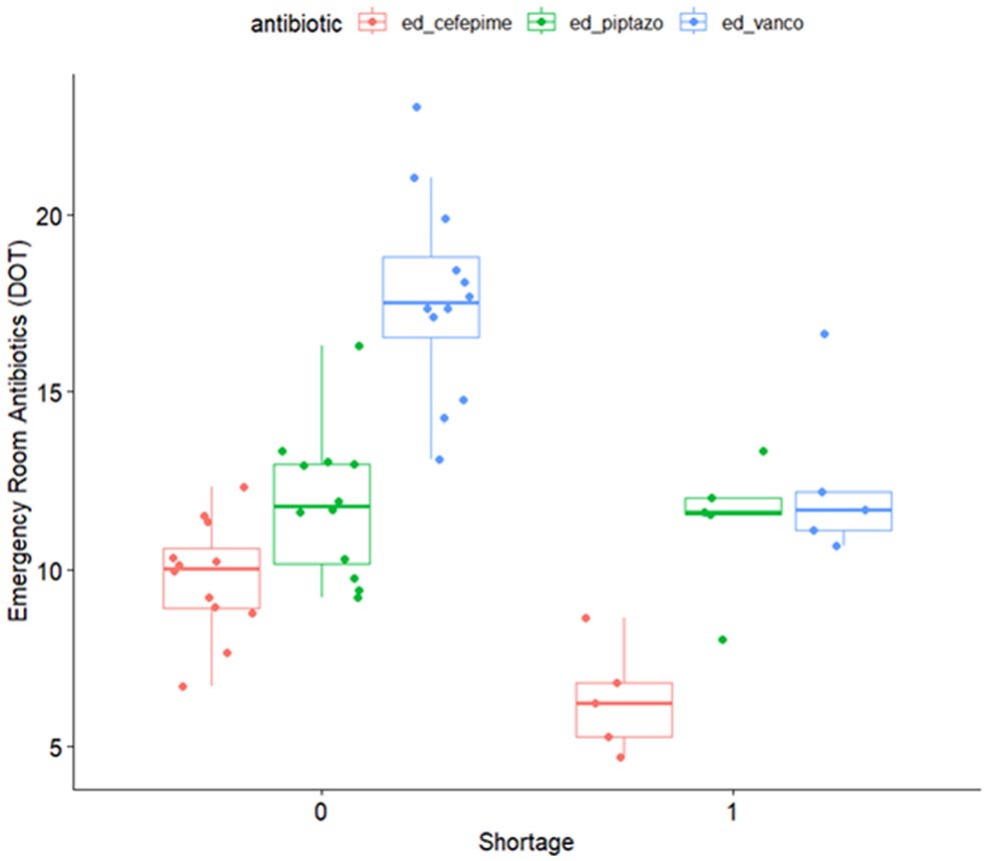
Box plots of antibiotic use in the emergency department for the compared time periods

**Methods:**

A retrospective pre-/post- study compared parameters pre-shortage (7/2023-6/2024) to the shortage period (7/2024-11/2024) at a large academic medical center. Shortage mitigation included: electronic clinical decision support (CDS) offered soft stops on repeat blood cultures and notified clinicians of the shortage via a best practice advisory, requiring acknowledgement of repeat BCx done within 48 h and offering alternative cultures. Extensive education was conducted, with specific outreach to high use departments such as the emergency department (ED) and Oncology floors. The primary outcome was days of antibiotic therapy (DOT) per 1000 patient-days using National Healthcare Safety Network data. Secondary outcomes included BCx utilization, SEP-1 compliance, length of stay (LOS), inpatient mortality, and BCx contamination rate. The Wilcoxon rank sum test was used to compare the median values between pre-shortage and shortage.

**Image 3:**
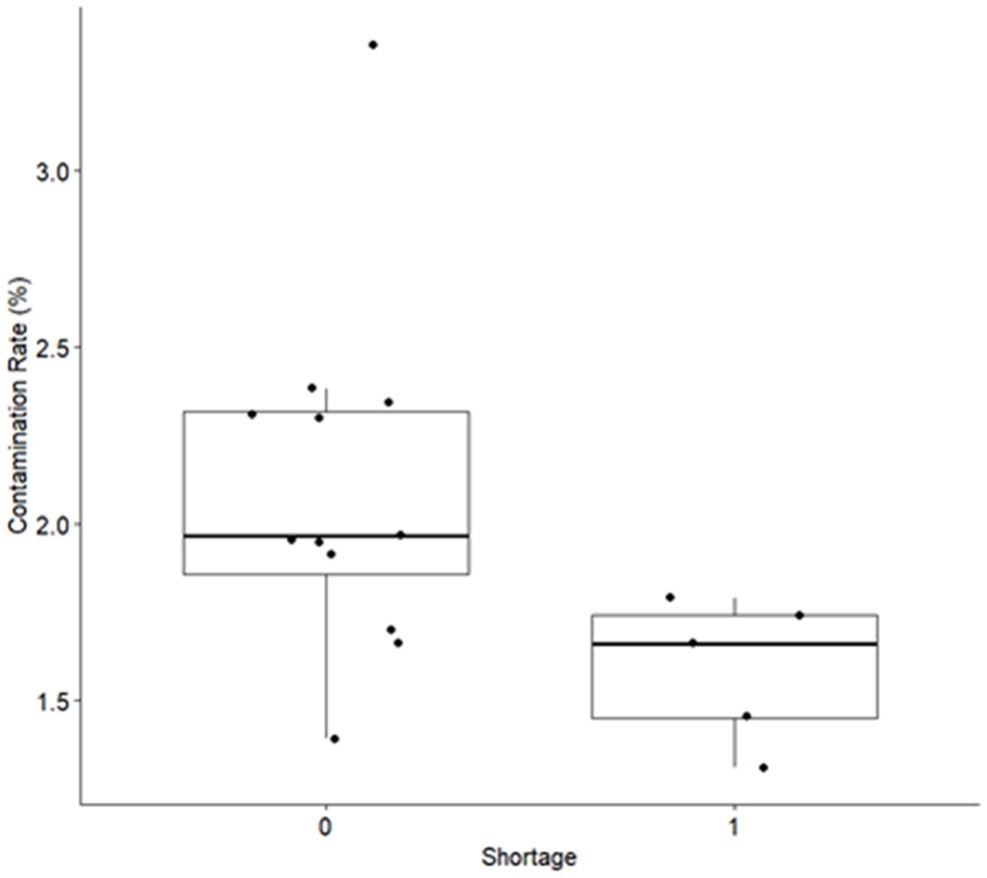
Box plots of percent blood culture contamination rate for the compared time periods

**Table 1:**
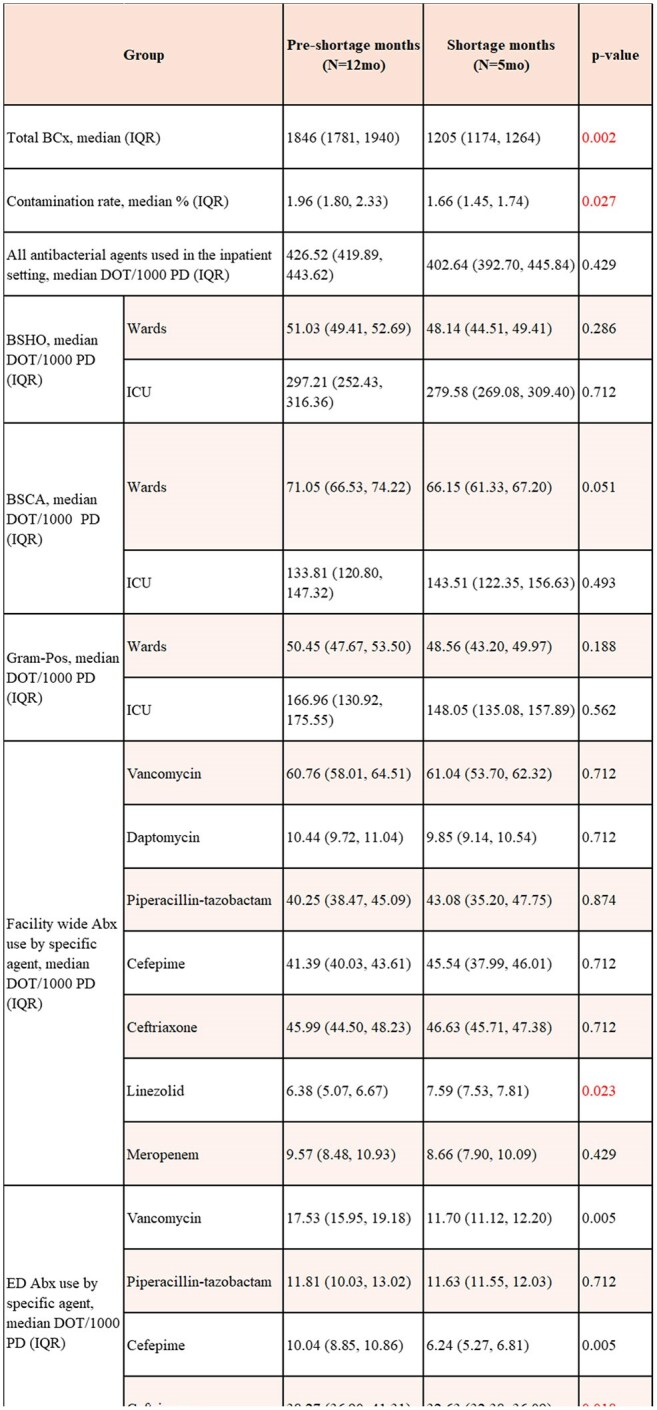
Results summary with comparison of time periods for number of cultures obtained, antibiotic use, contamination rates, length of stay, inpatient mortality, and core sepsis measures Abbreviations: BSHO: broad-spectrum antibacterial agents for hospital-onset infections; BSCA: broad-spectrum antibacterial agents for community-acquired infections; DOT: days of therapy; ED: emergency department; Gram-Pos: antibacterial agents for resistance gram-positive infections; ICU: intensive care unit; IQR: interquartile range; LOS: length of stay; SEP-1: Severe Sepsis and Septic Shock Management Bundle

**Results:**

Comparison of the time periods is shown in Table 1. BCx utilization decreased during the shortage from a median of 1846 to 1205 (p=0.002), visualized by box plot in image 1. Overall antibacterial (Abx) use did not change nor did use by NHSN category. When evaluated by location, ED vancomycin use declined from a median of 17.53 to 11.70 DOT/1000 patient days (p=0.005), cefepime from 10.04 to 6.24 (p=0.005), and ceftriaxone from 38.27 to 32.63 (p=0.018), visualized in Image 2. Mortality did not change, but length of stay decreased mildly from 7.01 to 6.71 mean days observed (p=0.013). Contamination rates decreased from 1.96 to 1.66 (p=0.02), visualized in Image 3.

**Conclusion:**

While concern existed that decreased BCx use may result in increased empiric antibiotic use, we found no change in antibiotic use during the shortage. Interestingly, significant decreases in some antibiotics were noted in the ED. These findings suggest that while the shortage influenced certain aspects of antibiotic prescribing and contamination rates, it did not adversely affect overall sepsis management and patient outcomes.

**Disclosures:**

Trevor C. Van Schooneveld, MD, FSHEA, FIDSA, Mannki: Grant/Research Support

